# A metabolomics characterisation of natural variation in the resistance of cassava to whitefly

**DOI:** 10.1186/s12870-019-2107-1

**Published:** 2019-11-27

**Authors:** Laura Perez-Fons, Adriana Bohorquez-Chaux, Maria L. Irigoyen, Danielle C. Garceau, Kris Morreel, Wout Boerjan, Linda L. Walling, Luis Augusto Becerra Lopez-Lavalle, Paul D. Fraser

**Affiliations:** 1 School of Biological Sciences, Royal Holloway University of London, Egham, UK; 2 International Center of Tropical Agriculture (CIAT), Cali, Colombia; 3 Department of Botany and Plant Sciences, University of California, Riverside, California, USA; 4 Ghent University, Department of Plant Biotechnology and Bioinformatics, Technologiepark 71, 9052 Ghent, Belgium; 5 VIB Center for Plant Systems Biology, Technologiepark 71, 9052 Ghent, Belgium

**Keywords:** Cassava, Whitefly, Metabolomics, LC-MS, Resistance, Phenylpropanoids, Lignin

## Abstract

**Background:**

Cassava whitefly outbreaks were initially reported in East and Central Africa cassava (*Manihot esculenta* Crantz) growing regions in the 1990’s and have now spread to other geographical locations, becoming a global pest severely affecting farmers and smallholder income. Whiteflies impact plant yield via feeding and vectoring cassava mosaic and brown streak viruses, making roots unsuitable for food or trading. Deployment of virus resistant varieties has had little impact on whitefly populations and therefore development of whitefly resistant varieties is also necessary as part of integrated pest management strategies. Suitable sources of whitefly resistance exist in germplasm collections that require further characterization to facilitate and assist breeding programs.

**Results:**

In the present work, a hierarchical metabolomics approach has been employed to investigate the underlying biochemical mechanisms associated with whitefly resistance by comparing two naturally occurring accessions of cassava, one susceptible and one resistant to whitefly. Quantitative differences between genotypes detected at pre-infestation stages were consistently observed at each time point throughout the course of the whitefly infestation. This prevalent differential feature suggests that inherent genotypic differences override the response induced by the presence of whitefly and that they are directly linked with the phenotype observed. The most significant quantitative changes relating to whitefly susceptibility were linked to the phenylpropanoid super-pathway and its linked sub-pathways: monolignol, flavonoid and lignan biosynthesis. These findings suggest that the lignification process in the susceptible variety is less active, as the susceptible accession deposits less lignin and accumulates monolignol intermediates and derivatives thereof, differences that are maintained during the time-course of the infestation.

**Conclusions:**

Resistance mechanism associated to the cassava whitefly-resistant accession ECU72 is an antixenosis strategy based on reinforcement of cell walls. Both resistant and susceptible accessions respond differently to whitefly attack at biochemical level, but the inherent metabolic differences are directly linked to the resistance phenotype rather than an induced response in the plant.

## Background

Cassava (*Manihot esculenta* Crantz) is a woody shrub that is native to South America, which was originally domesticated in the Amazon basin. Cassava was first introduced into Africa during the 1500s, where it evolved into a staple food source, and got widely distributed across tropical regions during the 18th and nineteenth century [1]. Several key attributes have contributed to cassava as a food source in these regions; they include its ability to grow on marginal land with poor soil parameters and its high starch content providing dietary caloric value. However, micronutrient content is low in root products [2], and this has led to the development of bio-fortification programs to alleviate micronutrient deficiency in developing countries [3].

More than 800 million people worldwide depend on cassava roots as a staple crop [4]. World production was estimated as > 290 million tonnes in 2017 (FAOSTAT, http://www.fao.org/faostat/en/#data/QC/visualize). Production in Africa and Central and South America is predominantly directed towards foodstuffs for human consumption, whilst the growing Asian markets dominate the export of cassava for industrial utilisation such as starch and biofuels.

More recently it has become evident that domesticated germplasm has not been adequately robust to cope with emerging abiotic and biotic stresses, which are proving a major threat to cassava production by smallholder farmers. For example, the African cassava mosaic (ACMV) and cassava brown streak (CBSV) family of viruses are the top damaging agents described [5]. ACMV is transmitted either by infected cuttings or by its vector, the whitefly *Bemisia tabaci*. Severe ACMV outbreak, like the one in the mid-1990s, caused total loss of the crop in parts of Kenya and Uganda, and up to 90% losses in India and Sri Lanka [4]. Very recently, the presence of the Sri-Lankan cassava mosaic virus (SLCMV) was reported for the 1st time in KaunMoum, Cambodia [6]. Today, a large proportion of cassava production areas in Cambodia and Vietnam shows high incidence of this disease likely transmitted by whiteflies; potentially rendering its production unsustainable in this region.

Despite several crop management recommendations, super abundant whitefly outbreaks and associated virus outbreaks occur on a regular basis in these sensitive regions. One approach to address whitefly infestation as the cause of crop wastage is through the development of resistant or tolerant cassava varieties to the whitefly. Societal concerns over Genetic Modification (GM) technologies means that the exploitation of natural variation is paramount to crop improvement programs for both input and output traits. Natural sources of whitefly resistance have been identified in germplasm collections held in the International Center of Tropical Agriculture (CIAT) and the International Institute of Tropical Agriculture (IITA), covering wild relatives species and landrace accessions [7–10]. CIAT’s accession ECU72 consistently demonstrated resistance to the South American whitefly *Aleurotrachelus socialis* and recently to the African whitefly *B. tabaci* as it showed reduced oviposition rates and adults’ preference, and higher nymph mortality. ECU72 also presented lower damaging scores upon whitefly infection, an indication of tolerance to biotic stress [11]. Other landrace accessions within the CIAT genebank have also been reported to display tolerance and/or resistance to whitefly species [8, 10].

To date, how these accessions confer tolerance and/or resistance to whitefly infestation at the molecular and biochemical levels awaits elucidation. Such advances in our knowledge are essential to augment existing and future cassava breeding programs directed towards the development of biotic stress resistant varieties. The current genetic resources available for cassava have substantially driven research towards development of resistant varieties to ACMV and CBSV or both, such as genomic selection and marker-assisted selection [12]. Concomitantly, transgenic approaches have also produced virus resistant plants, but deployment to fields is pending on regulatory approval. Nevertheless, the whitefly vectoring role has received little attention, and studies on understanding the biology of the insect and identification and development of resistant lines have only recently been initiated (www.cassavawhitefly.org). In comparison to commercial crops consumed in western societies, genetic resources and tools are a limiting factor in cassava breeding. Despite noticeable advances in cassava genomics [13, 14], complementary “omic” technologies remain poorly utilised in assisting cassava’s crop improvement programs. In the present work, a metabolomics approach has been used to investigate the resistance/tolerance mechanisms to whitefly in cassava by comparing a resistant (ECU72) and a susceptible (COL2246) variety. The findings are discussed with respect to the rational development of pre-breeding materials for the implementation of whitefly resistant cassava varieties in Sub-Saharan Africa.

## Results

### Generation of resistant and susceptible cassava leaf material to *Aleurotrachelus socialis*

**Fig. 1:**
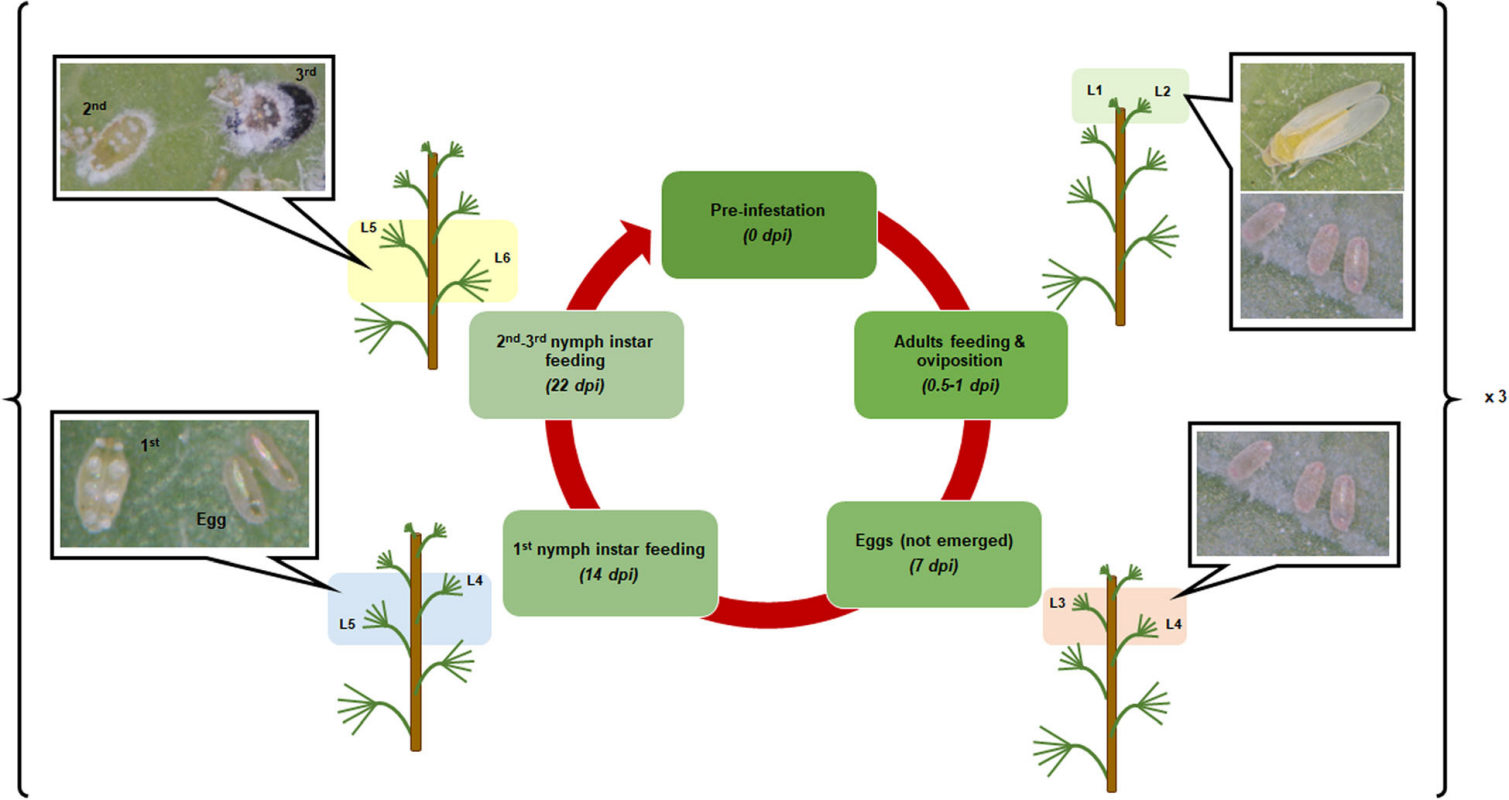
The generation of cassava leaf material infested with *Aleurotrachelus socialis****.*** Five plants per time-point and genotype were incubated in enclosed cages for 3 days with whitefly colonies. After eggs were laid out during incubation time (72 h), whitefly adults were released and plants taken out of the cages and transferred to a whitefly-free environment to allowed progression of whitefly cycle and prevent recurrent infestation of emerging leaves. Coloured boxes highlight those leaves collected for metabolomics analysis and where whiteflies developed. Three replicates of non-choice infestation trials were performed and analysed independently. Dpi: days post-infestation; L: number of leaf counting from top emerging leaf. Pictures taken by A.B.C and L.A.B.L-L at CIAT

In order to apply our investigative comparative metabolomics approach, non-infested and infested cassava leaf material was generated. Cassava plantlets were grown to a developmental stage whereby the first five leaflets were expanded. The first two expanded leaves of five biological replicates were harvested representing time-point 0 (T0), and concurrently over the life cycle of the whitefly, leaves were collected at 12 h, 24 h, 7 days, 14 days and 22 days post-infestation for both COL2246 and ECU72 independently exposed to *A. socialis* colonies. Both varieties were exposed to identical population size of whitefly adults. The infestation trials performed in the present work were dedicated to evaluate the susceptibility or resistance of both genotypes as means of ability to reduce infection. Evaluation of tolerance as yield loss or plant fitness was out of the scope of the present study. ECU72 presented a significant reduced number of eggs deposited on leaves when compared to COL2246 (Additional file 1: Figure S1) validating its resistant phenotype. A complementary experiment was run in parallel in absence of whitefly. Leaves at progressive developmental stages and free of whitefly were collected and used as mock-infestation control in order to exclude whitefly metabolites contamination and leaf development effects. This material was then prepared for comparative metabolomics using LC-MS and GC-MS metabolite profiling approaches. An illustrative representation of the sampling methodology is provided in Fig. 1.

### Cassava leaf’s metabolome

Using complementary LC-MS and GC-MS metabolite profiling built upon the non-targeted approach enabled the unambiguous structural characterization of 184 components and 28 level 4-unknowns according to the Metabolomics Standard Initiative [15] (Additional file 2: Table S1). This matrix of data containing the annotated and characterized metabolites was used as targeted dataset. The compounds characterized at T0 (pre-infestation) in both COL2246 and ECU72 were also present across the multiple infestation time-points and no additional metabolites detected in the infested leaves were included in the targeted dataset to prevent ambiguous interpretation of data as they could originate from whitefly metabolome. Compounds characterized by LC-MS comprised a range of secondary metabolites dominated by the presence of phenylpropanoids and flavonoids and certain compounds related to primary metabolism, e.g., amino acids and mono- and disaccharides. Phenylpropanoids including ester-derivatives of hydroxycinnamic acids along with related monolignols and oligolignols [16, 17] were evident. Chemical variation was also extended to additional classes of compounds involving cyanogenic glycosides, hydroxybenzoates and glycosylated apocarotenoids (Additional file 2: Table S1). The GC-MS analysis of polar extracts facilitated the annotation of components of intermediary/primary metabolism. For example, components of TCA cycle and glycolysis, mono- and disaccharides, alcohol and acid sugars, amino acids and polyamines. Analysis of non-polar extracts by gas chromatography allowed detection of triterpenoids, in either their free or glycosylated forms, tocopherols, fatty acids or alkanes.

### Identification of changes in the cassava metabolome upon exposure to *A. socialis*

**Fig. 2:**
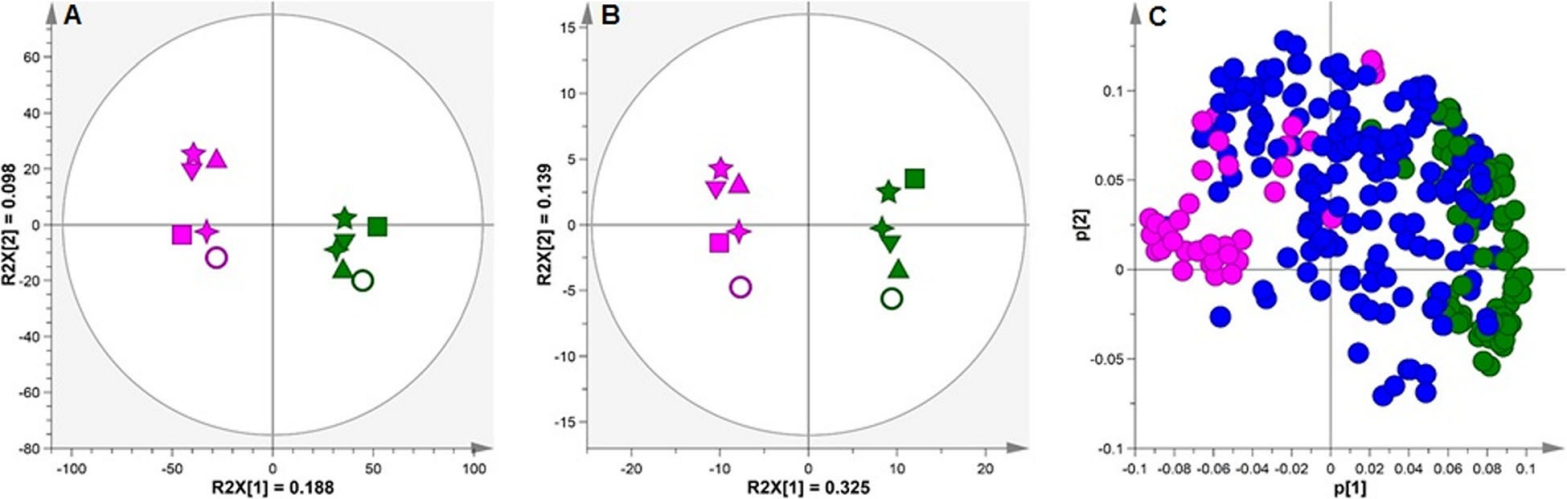
Component 1 and 2 score plots of principal component analysis of (**a**) Non-targeted LC-MS analysis, and (**b**) LC-MS targeted analysis and (**c**) loadings plot of LC-MS targeted analysis where significant (*p* < 0.05) features altered in ECU72 (pink) and COL2246 (green) are highlighted. Green and pink symbols represent infestation time-points of susceptible variety COL2246 and resistant variety ECU72, respectively. Collection times during infestation were defined by the following symbols: **◯** 0 days post-infestation (T0); ▼ 0.5 day (12 h) post-infestation (T1); ▲ 1 day post-infestation (T2); ■ 7 days post-infestation (T3); ✦ 14 days post-infestation (T4) and ★ 22 days post-infestation (T5). Principal component analysis plots were performed using Simca software and using pareto-scaling method. Averaged biological and technical replicates are presented to facilitate visualisation

Non-targeted analysis by LC-MS revealed 9287 chemical features (Additional file 3: Table S2) which when analysed by Principal Component Analysis (PCA) rendered separation of the genotypes regardless of infestation or duration of infestation. In this instance, the score plot of components 1 and 2 (Fig. 2a) explained 18.88% of the variability, whereby genotypes separated along the PC1 axis (18.8% variability) and time-points along the PC2 axis (0.098% variability). The targeted profiling approaches based on 212 features enabled more robust quantification and characterization of key metabolites across important sectors of metabolism. When incorporated into PCA, a similar clustering pattern of genotypes was revealed (Fig. 2b) with PC1 and PC2 explaining 32.5 and 13.9% of the variability, respectively. PCA performed on GC-MS data yielded similar results (Additional files 4: Figure S2 and 5: Figure S3). Collectively, the untargeted analysis in combination with the targeted metabolite profiling revealed clear chemical differences in the metabolomes of COL2246 and ECU72, regardless of the infestation treatment and its progression. The characterization of metabolites in high confidence also facilitated data mining for key biochemical differentiators between the two genotypes, leaf development and responses to *A. socialis* infestation.

### Differentiating chemical signatures between the susceptible and resistant genotypes

#### Changes in phenylpropanoid

**Fig. 3:**
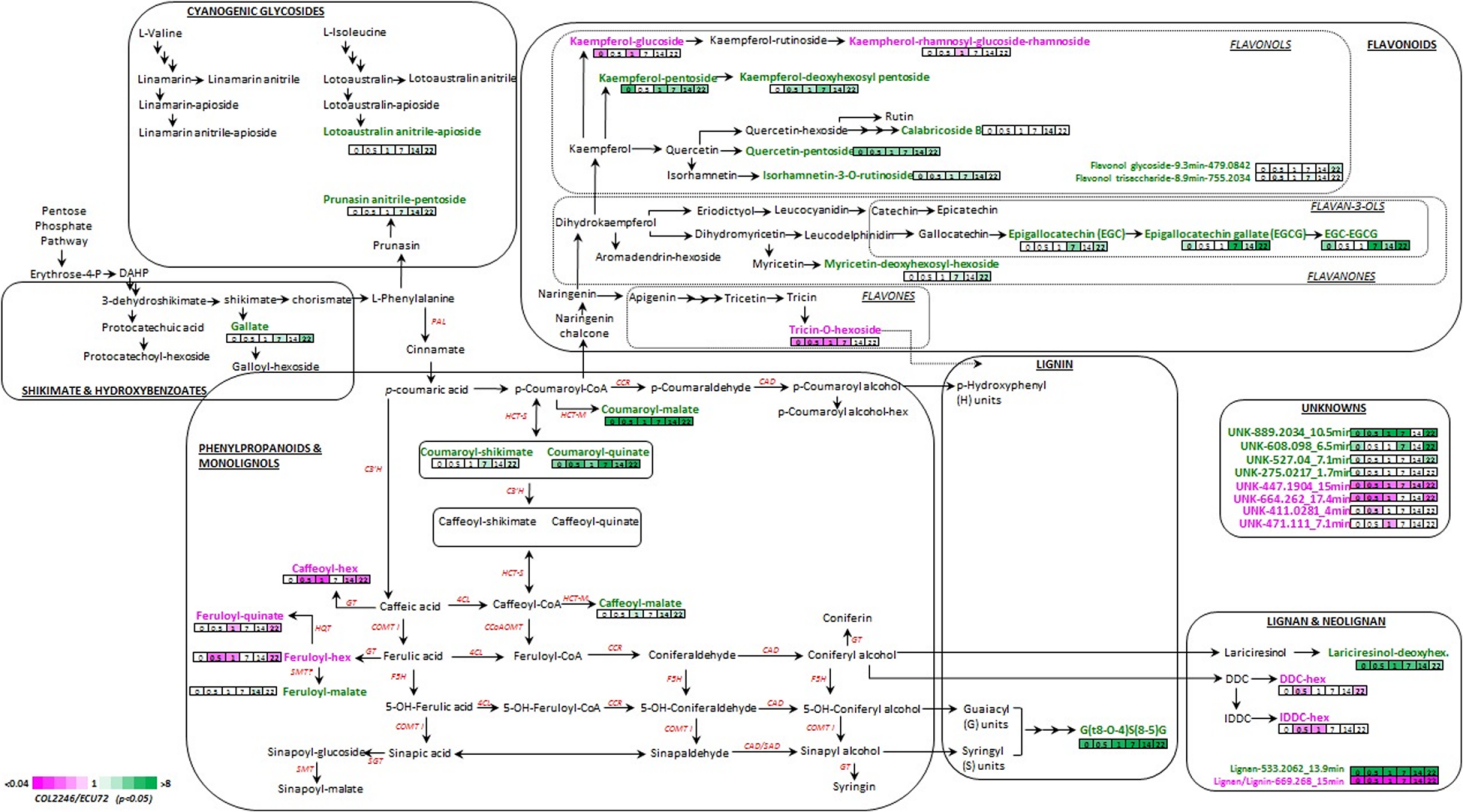
Pathway display visualisation of significant changes in secondary metabolite abundances observed between COL2246 and ECU72 during the infestation cycle. Cells indicate time-points and were coloured according to their respective fold-change, green cell indicating significant accumulation of corresponding metabolite in susceptible variety COL2246 and pink cells representing significant increased levels of metabolites in resistant variety ECU72 respective COL2246

Pair-wise comparison of both genotypes at every time-point of the infestation cycle revealed the following quantitative differences in metabolite’s composition: *p*-coumaroyl esters of shikimic acid, quinic acid and malic acid accumulated significantly (*p* < 0.05) in the susceptible variety COL2246 along with malate esters of caffeic acid and ferulic acid (Fig. 3; Additional file 2: Table S1). Glycosylated forms of the flavonols kaempferol and quercetin occurred in both genotypes. However, the pentose-derivatives preferentially accumulated in COL2246 and the hexose derivatives in ECU72. Flavan-3-ols epigallocatechin (EGC), epigallocatechin gallate (EGCG) and its corresponding dimer EGC-EGCG were significantly higher in abundance in the susceptible genotype COL2246 when compared to the resistant ECU72. In addition, the trihydroxybenzoate gallic acid incorporated into these molecules also had a higher abundance in COL2246. Another family of compounds that presented higher levels in the susceptible variety were identified as pentoside derivatives of the cyanogenic glycosides prunasin and lotoaustralin in their anitrile (non-active) forms. The oligolignol G(*t*8–O–4) S(8–5)G [18] and the lignans lariciresinol-deoxyhexoside and two non-identified components were also consistently higher in COL2246. From the MS spectra it could be deduced that these two unknown compounds are related to lignan. For example, the unknown feature with a retention time at 13.9 min and m/z value of 533.2062 generated a chemical formula of C27H34O11 (Additional file 6: Table S3) that retrieved 75 and 3 hits when blasted against the chemical database Chemspider and ChEBI, respectively. Twenty out 75 entries retrieved from Chemspider matched with the lignan arctiin and 17 out 75 Chemspider’s entries matched the lignan identified as phyllirin (or forsythin), an 8,8’-coupled lignan similar to lariciresinol that also accumulated in COL2246 in its glucosylated form. Similarly, an unknown compound at 10.5 min and having an accurate m/z value of 889.2034 (UNK-889.2034-10.5 min) showed a similar accumulation profile in the infected leaves of COL2246 as those of the oligolignols and putative lignans detected in the present work, suggesting it could be a structurally related molecule (Fig. 3; Additional file 6: Table S3).

In contrast, the resistant variety ECU72 displayed significantly higher levels of hexoside derivatives for a range of compounds such as caffeic acid, ferulic acid, the neolignans dehydrodiconiferyl alcohol (DDC) and isodehydrodiconiferyl alcohol (IDDC), and the flavonoids tricin and kaempferol. In addition, a number of unknown metabolites were also observed in ECU72 that might represent (neo)lignans/oligolignols based on their accurate mass (Fig. 3; Additional file 6: Table S3).

The differential accumulation of certain compounds in COL2246 was noticeable over the course of the whitefly infestation cycle and already present at pre-infestation stage (0 dpi). For example, coumaroyl-quinate and malate esters, oligolignol G(*t*8–O–4) S(8–5)G, lignans lariciresinol-deoxyhexoside, putative UNK-533.2062-13.9 min and UNK-889.2034-10.5 min presented a fold-change increase in COL2246 higher than 4, and the levels of flavonoids quercetin pentoside and isorhamnetin-3-O-rutinoside in the susceptible leaves of COL2246 were > 2 fold-change higher prior to and during infestation (Additional file 2: Table S1). Pentoside derivatives of kaempferol were also consistently higher during the time-course of the infestation from 0.5 dpi onwards (Additional file 2: Table S1), and accumulation of epigallocatechins and gallic acid were also significantly higher in COL2246 at late stages of infestation (≥7 dpi). In the resistant genotype ECU72, the hexoside derivatives of flavonoids and lignans were significantly higher at early stages of the time-course experiment, i.e. < 7 dpi, when compared to susceptible COL2246, whereas the phenylpropanoids caffeoyl and feruloyl-hexosides were also significantly increased at 22 dpi (Fig. 3; Additional file 2: Table S1).

#### Differences between genotypes in sterols composition and content upon whitefly infection

**Fig. 4:**
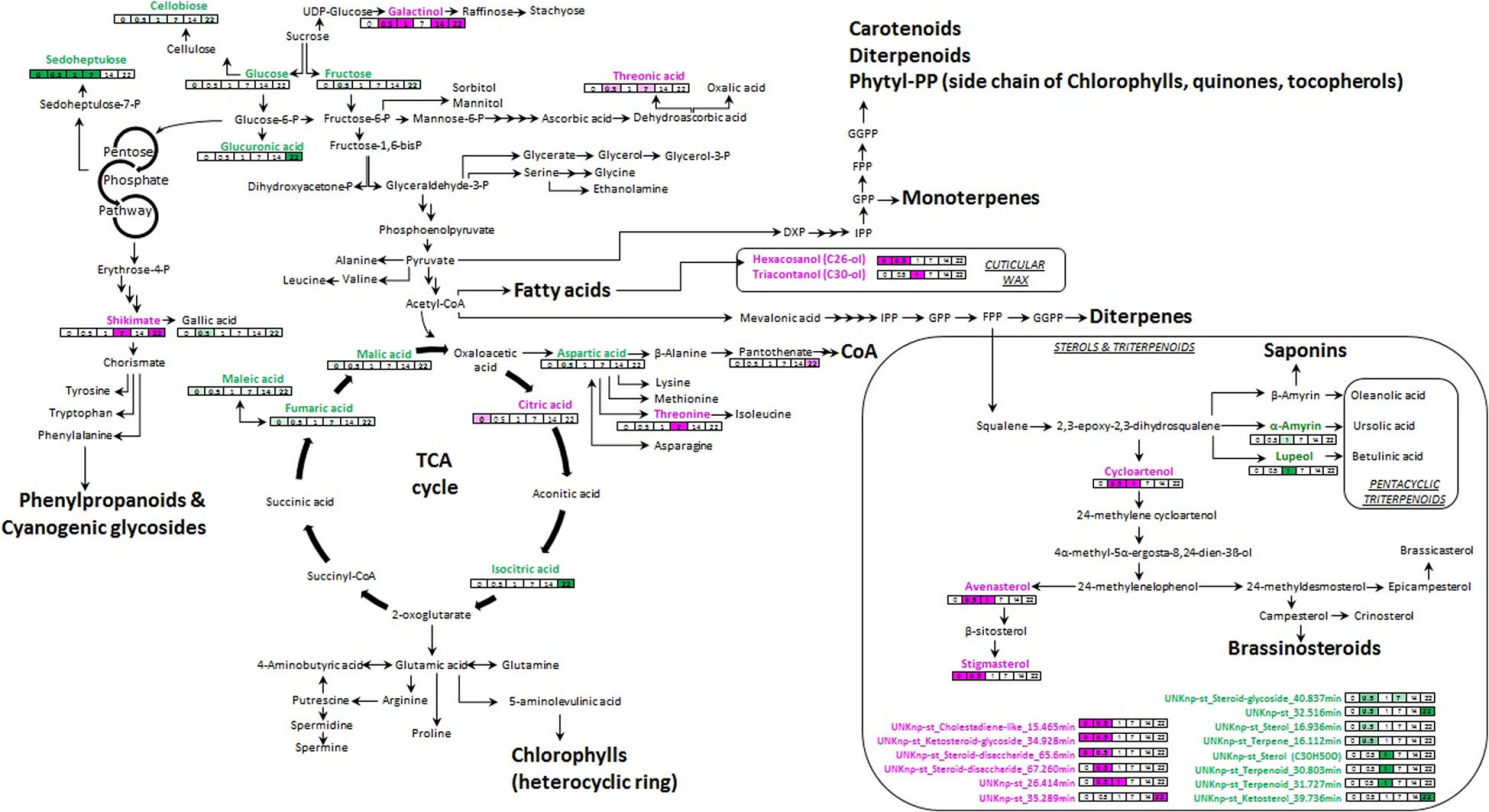
Pathway display visualisation of significant changes in primary/intermediary metabolite abundances observed between COL2246 and ECU72 during the infestation cycle. Cells indicate time-points and were coloured according to their respective fold-change, green cell indicating significant accumulation of corresponding metabolite in susceptible variety COL2246 and pink cells representing significant increased levels of metabolites in resistant variety ECU72 respective COL2246

Although the differences between genotypes in the phenylpropanoid content were the overriding comparative features of the metabolomes (Fig. 3), alterations in the levels of sterols, triterpenoids and wax components (long chain alkanes) were also evident, as displayed across their respective biosynthetic pathway display (Fig. 4). These significant differences between genotypes occurred at early stages of infestation (< 7 dpi) or were already present before exposure to whitefly (0 dpi). Two non-identified compounds UNK_np-st__Ketosterol_39.736 min and UNK_np-st__32.516 min were the exception as their abundances were significantly higher in COL2246 at 22 days post-infestation, respectively.

The long-chain alcohols hexacosanol and triacontanol and the sterols cycloartenol, avenasterol and stigmasterol preferentially accumulated in the resistant variety ECU72 at early stages. A group of non-identified steroid-glycosides showed a similar pattern of accumulation while the abundance of the triterpenoids α-amyrin and lupeol and unknown sterols and terpenoid metabolites were significantly higher in the susceptible variety COL2246.

A significant proportion of the sterol molecules could not be resolved with the experiments conducted in the present work. It was clear from their EI (GC-MS) spectrum that they were present as glycosylated forms but references regarding the identification of this class of compounds in cassava are scarce [19, 20]. Therefore, a detailed characterisation of this particular class of compounds is required to further elucidate their structures but is not the scope of the present work.

#### Differences in intermediary metabolism

Subtle changes were observed in the primary metabolism components mainly linked with carbohydrate metabolism. The susceptible variety COL2246 had higher levels of the monosaccharides glucose, fructose and glucuronic acid, the heptose sedoheptulose and the disaccharide cellobiose. The abundance of the tricarboxylic acid cycle (TCA cycle) components was also higher in COL2246 (Additional file 2: Table S1, Fig. 4) whilst the resistant variety ECU72 showed higher amounts of threonic acid and galactinol, citric acid and the amino acid threonine (Fig. 4).

#### Temporal variation of cassava metabolome upon *A. socialis* infestation

Analysis of variance was applied independently to time-course experiments of COL2246 and ECU72 (Additional files 7, 8: Tables S4, S5). Those metabolites displaying significant (*p* < 0.05) changes under multiple statistical post-hoc tests were selected for further discussion.

**Fig. 5:**
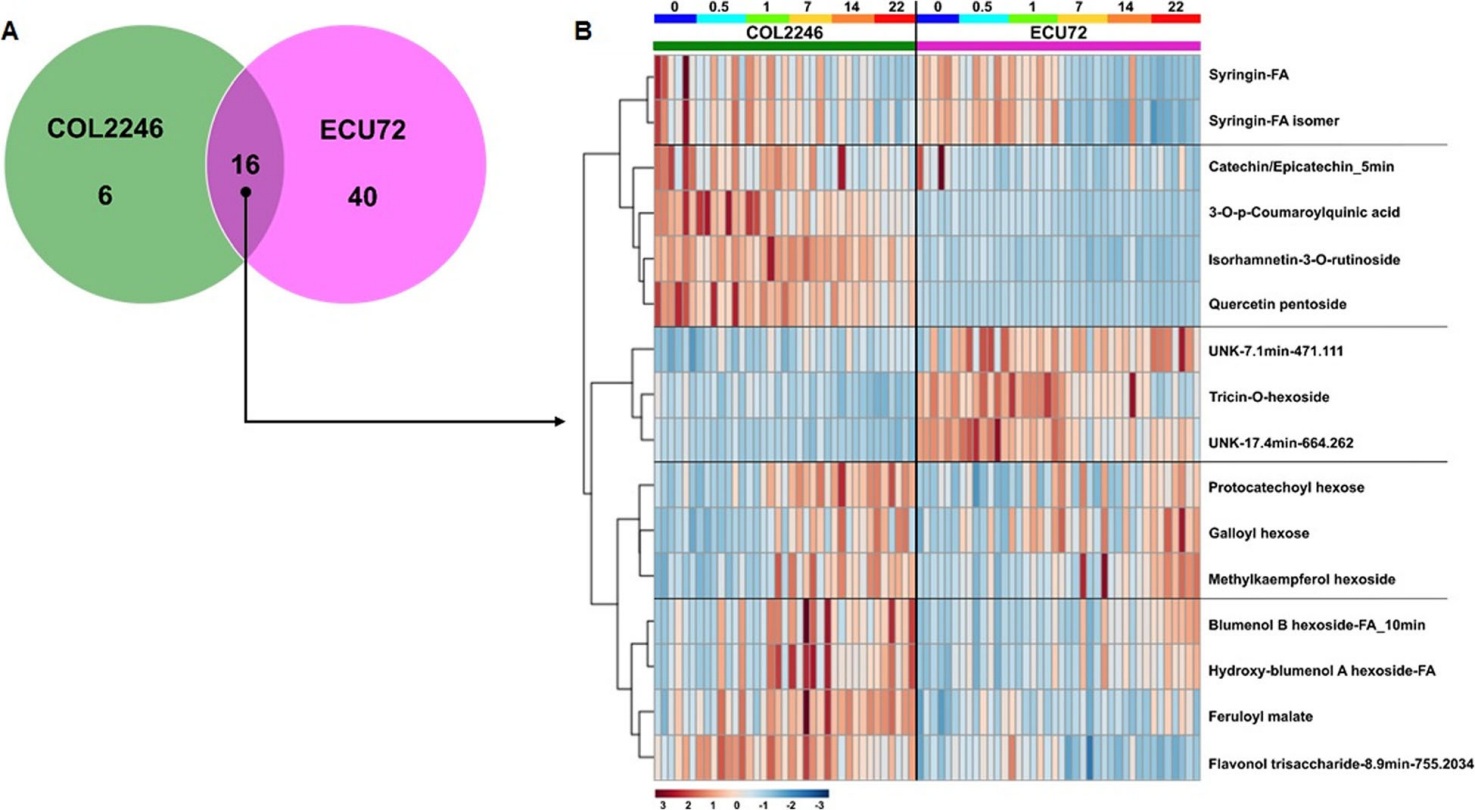
**a** Venn diagram of features significantly (*p* < 0.05) varying during the infestation time course in COL2246 and ECU72. **b** Heat-map and temporal variation of core metabolites changing over time in both COL2246 and ECU72. Infestation time points indicated as days post-infestation (dpi)

The ANOVA results summarized in Fig. 5a revealed 56 metabolites that significantly changed over time in ECU72. Similarly, 22 metabolites varied with infestation progression in COL2246, 16 of which were the same as in ECU72.

Changes over time in this core group of 16 metabolites are displayed as a heat map (Fig. 5b) where at least five differential patterns can be observed when comparing both genotypes COL2246 and ECU72. The first cluster defined by syringin and its isomer indicated similar levels and temporal variation in both genotypes during infestation. Clusters 2 and 3 were defined by metabolites that reciprocally accumulate in both varieties, i.e., preferentially accumulated in COL2246 or ECU72, respectively. Both clusters of metabolites generally presented similar variation with time with subtle exceptions (Additional file 9: Figure S4). For example in cluster 2, the flavonol isorhamnetin-3-O-rutinoside increased in COL2246 but decreased in ECU72 at 1 and 7 dpi; or the UNK-7.1 min-471.111 in cluster 3 which tended to decrease from 0.5 to 14 dpi in the susceptible variety COL2246 and increased over time in the resistant ECU72. The di- and tri- hydroxybenzoates protocatechoyl and galloyl hexosides and the flavonol methylkaempherol hexoside constituted cluster 4 of metabolites and had similar levels in both genotypes but contrasting accumulation at 14 dpi. Similarly, the last cluster represented metabolites consistently accumulating in the susceptible variety COL2246 and gradually increasing with infestation progression in both varieties, except at 7 dpi when a dramatic increase in the susceptible variety occurred followed by a decrease in the next time point. This subgroup of metabolites includes apocarotenoids, feruloyl malate and flavonol trisaccharide (8.9 min-755.2034). The temporal variation of some of these metabolites matched the variation observed during leaf development in the absence of whitefly infestation, suggesting that they could be linked to progression in leaf development rather than to response to infestation (Additional file 9: Figure S4). Some examples are syringin, the flavonols isorhamnetin-3-O-rutinoside and quercetin pentoside and the unknowns in cluster 3, i.e. UNK-7.1 min-471.111 and UNK-17.4 min-664.262.

**Fig. 6:**
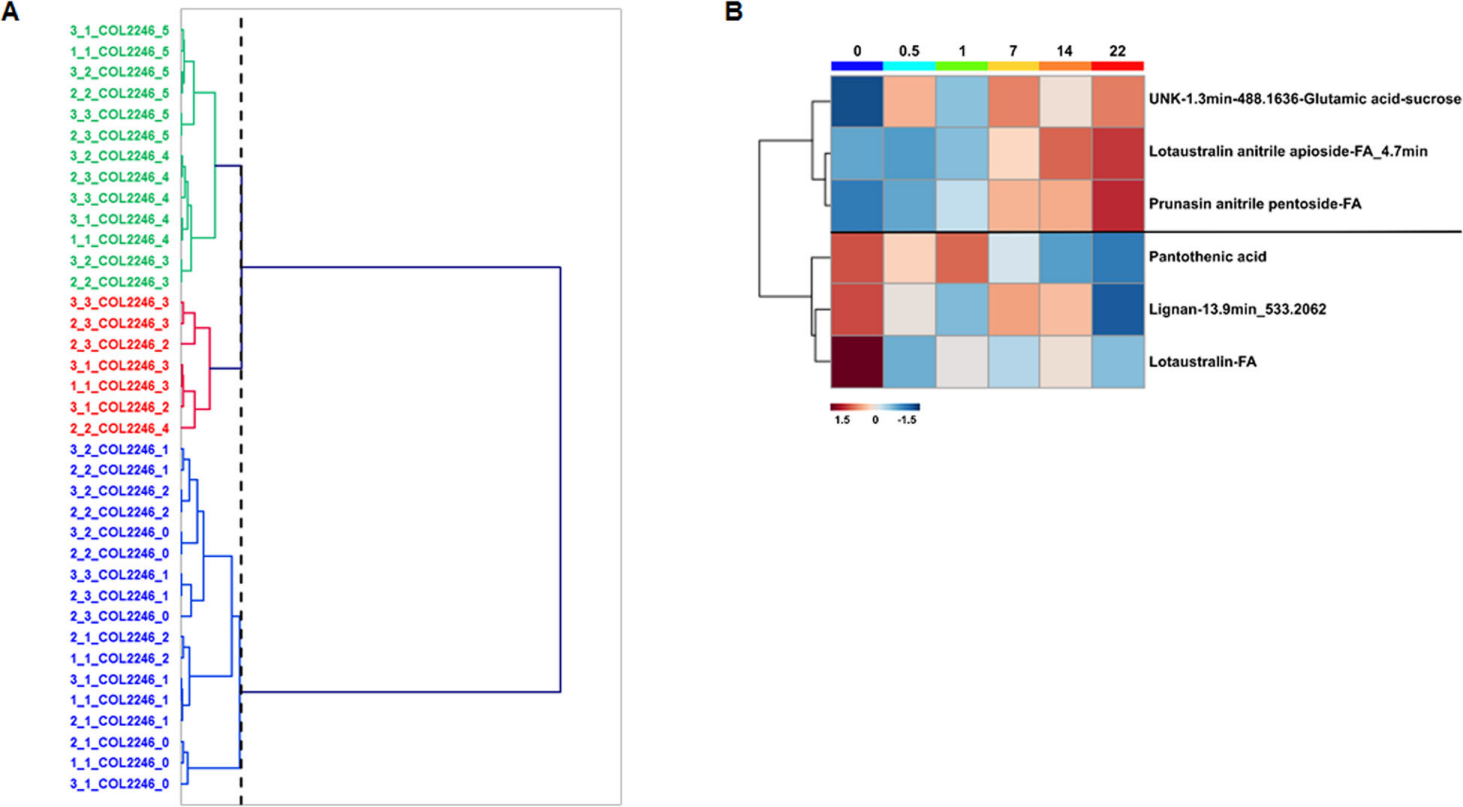
**a** Ward’s Agglomerative Hierarchical Clustering of infestation time-points using LC-MS targeted data in COL2246 and (**b**) Heat map of metabolites significantly changing over time in COL2246 exclusively. The dotted line in the dendrogram indicates the truncation level automatically generated by the software XLSTAT

**Fig. 7:**
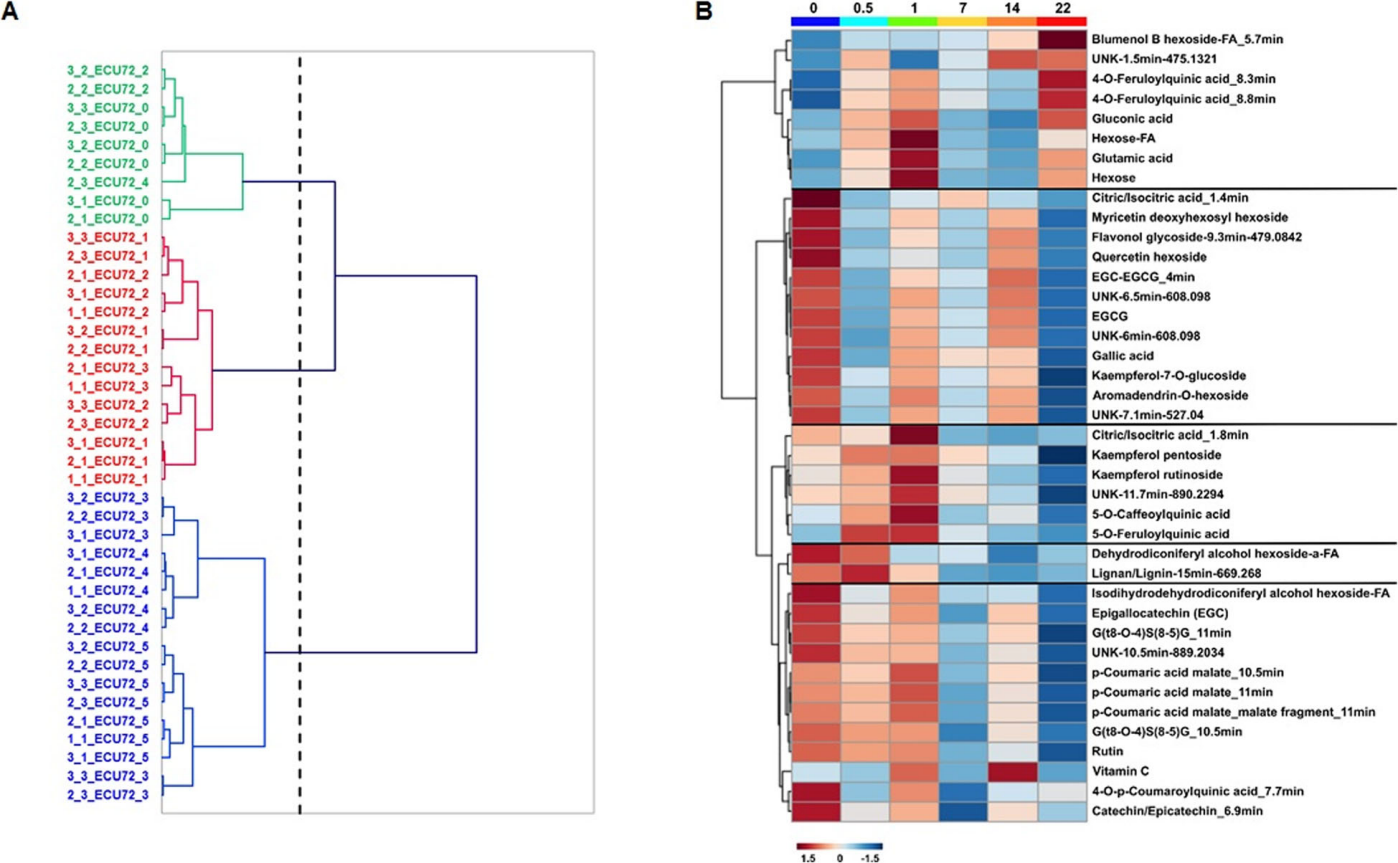
**a** Ward’s Agglomerative Hierarchical Clustering of infestation time-points using LC-MS targeted data in ECU72 and (**b**) Heat map of metabolites significantly changing over time in ECU72 exclusively. The dotted line in the dendrogram indicates the truncation level automatically generated by the software XLSTAT

Dendrogram classification of infestation time-points based on the LC-MS targeted matrix differentiated three clusters in both COL2246 (Fig. 6a) and ECU72 (Fig. 7a) but with different distribution of the time-points within the clusters. In the susceptible variety COL2246, the early (0–24 hpi) and late stages (14–22 dpi) of infestation were grouped in separate clusters 1 and 3 whilst the time-point 7 dpi was located in cluster 2. However, in the resistant variety ECU72 pre-infestation time-point 0 dpi constituted a cluster in itself and early (12–24 hpi) and late (7–22 dpi) post-infestation stages formed two distinctive groups (Fig. 7a). The distinction between early and late infestation events in the susceptible variety COL2246 was also evident in the metabolic changes occurring exclusively in this genotype (Fig. 6b). The group of 6 metabolites exclusively changing in COL2246 were arranged in two clusters displaying reciprocal temporal variation. Anitrile non-active forms of cyanogenic glycoside lotaustralin and prunasin and the UNK-1.3 min-488.163 tended to increase as the infestation progressed and these compounds reached their highest concentration at late stages. The opposite was observed for those metabolites grouped in cluster 2. Here, lotaustralin in its active form and the compounds pantothenic acid and the putative lignan-13.9-533.2062 were highly abundant at pre-infestation and rapidly decreased in abundance during the time-course.

The clustering and varying levels of the 40 metabolites changing uniquely in ECU72 is shown in Fig. 7b. At least 5 clusters displaying different patterns of temporal variation were identified. The first cluster is the only one with a positive tendency, i.e., the metabolite’s levels increased with time, whereas the remaining 4 clusters decreased during infestation. Different fluctuation patterns along the time-course were distinguished in clusters 2 to 4. Cluster 2 presented the highest concentration at pre-infestation followed by rapid decrease as infestation progressed but showed peaks of increase at 1 and 14 days post-infestation. Cluster 3 was characterised by a temporal pattern of increasing concentration from pre-infestation up to 1 dpi followed by a rapid decrease over the later time points. Metabolites in cluster 4 and 5 maintained high levels at early infestation time-points up to 1 dpi and then progressively reduced in abundance with time, except for cluster 5 which showed a surge at 14 dpi.

## Discussion

### Hierarchical metabolomics as a means of assessing complex natural variation

The untargeted metabolomics approach used created a robust chemical fingerprinting of the cassava metabolome of the two genotypes in question. The overall variance facilitated the separation of the genotypes, from which chemical differentiators could be determined. More targeted metabolite profiling enabled us to incorporate greater robustness in our components and assign chemical identification to the key metabolites of interest. These approaches can also be informative to assess genetic drag arising from future introgressions into donor germplasm when pyramiding traits.

In general, the data represent one of the most comprehensive studies of cassava to date. Through the use of the hierarchical metabolomics approach, the study has characterized over 200 metabolites in cassava with high confidence across a wide dynamic range and representing key sectors of the metabolism. An initial biochemical network has been created which can be integrated with future complementary omic datasets, such as transcriptomics to create potential correlation networks. Such an approach will enable a systems levels comparison between the resistant and susceptible varieties; potentially revealing robust metabolite and molecular markers for the trait of interest.

### Potential biochemical mechanisms conferring whitefly resistance

Numerous examples exist of mutants/transgenic plants that are altered in the abundance of monolignol biosynthesis pathway intermediates [21–25], in a way reminiscent to the metabolic differences observed between COL2246 and ECU72. What are the implications of the elevated levels of the malate, quinate and shikimate esters of the hydroxycinnamic acids *p*-coumaric, caffeic and ferulic acid in the susceptible variety? The main hypothesis is based on an alteration of the lignification, based on a lower activity in the last steps of the monolignol biosynthesis pathway in the susceptible cassava. This may lead to the diversion of the monolignol precursors into their CoA-thioester forms that are rapidly converted to their respective malate, quinate, or shikimate esters by hydroxycinnamoyl transferases (HCT) which are able to transfer shikimate, quinate, and malate to hydroxycinnamoyl-CoA esters and vice versa [26–28]. For example, feruloyl-malate accumulates in *ccr*1-knockout mutants of *Arabidopsis thaliana* [22] at the expense of sinapoyl-malate, and hyperaccumulation of feruloyl and sinapoyl-malate esters as well as their glucosidic precursors occurs in *A. thaliana* mutants of the Mediator regulatory complex of lignin [29]. Nonetheless, it is likely that feruloyl-malate could be alternatively synthesised by transesterification of feruloyl-glucoside with no involvement of CoA ester intermediates as is the case in sinapoyl-malate biosynthesis [22, 30]. This may also explain the differential accumulation of these ferulate derivatives in ECU72 and COL2246. Complementary experiments of lignin analysis reinforce the current hypothesis as a higher content of lignin was present in the leaves of the resistant variety ECU72 (Additional file 10: Table S6). In addition, the different levels of ferulic acid and *p*-coumaric acid in the cell wall extracts (Additional file 10: Table S6) has an impact on the cell wall properties [31] that could be directly related to biomechanical defence as has been observed in other species [32].

Inhibition/activation by enzymatic products over their respective biosynthetic enzymes is a well-known regulatory mechanism of enzyme activity. For example, phenylalanine ammonium lyase (PAL), the first committed enzyme of phenylpropanoid biosynthesis, is inhibited by its product cinnamic acid [33]. In a similar way, the lack of lignin may re-activate the initial steps of the phenylpropanoid pathway to produce more monolignols. This hypothesis has been also suggested by other authors studying poplar and *Arabidopsis CCR*-downregulated plants where *PAL* transcript levels were elevated [23, 25]. In our case, this feedback is evident by the fact that the total amount of compounds derived from phenylalanine were significantly higher (up to 1.8-fold increase) in the susceptible variety COL2246 (Additional file 11: Table S7 and Additional file 12: Figure S5). This genotype presented increased amounts of hydroxycinnamic acids, flavonoids and cyanogenic glycosides when compared to the resistant variety ECU72, some of them being consistently high throughout the course of the infestation. The relative proportion of each chemical class of compounds remained unaltered within each genotype.

Surprisingly, hexoside derivatives (likely glucose) of hydroxycinnamic acids and certain flavonoids were more abundant in the resistant variety ECU72. Glucosides are traditionally considered as storage and transport forms of metabolites, and although the role of the different glycosyl transferases and membrane transporters remains controversial, it is clear that these compounds are involved in the homeostasis of monolignol metabolism and location [34].

Although a detailed in-depth analysis of carbohydrates hasn’t been performed, the GC-MS profile revealed a higher content of cellobiose in the susceptible variety COL2246. Cellobiose is a dimer of glucose and is a component of cellulose. Mutants of *CCR* in Arabidopsis showed decreased lignin content but no significant changes in carbohydrates [35].

Taking all these results into consideration plus the fact that PCA analysis indicated major differences between genotypes rather than between time-points of the infestation within a given genotype, we propose that the resistance strategy in ECU72 is based on an antixenosis mechanism associated with a reinforced lignified cell wall of vascular tissue preventing the whitefly feeding on the leaves of ECU72. Previous work on the feeding behaviour of glasshouse whitefly (*Trialeurodes vaporariorum*) on host recognition revealed that this phloem-feeding insect explores the mechanical resistance of the leaves by repeated injections of its stylet. This pre-feeding behaviour allows the fly to assess the rigidity/toughness of the leaves and to evaluate the accessibility to nutrients [36]. Oviposition rate positively correlated with the feeding behaviour being higher in the susceptible variety. Several articles on resistance mechanisms of other crops have also highlighted the antixenosis reinforced cell wall as the strategy for preventing whitefly or aphids attack.

#### Genotypes respond differently to whitefly infestation

Cassava accessions COL2246 and ECU72 experience a different response at the metabolic level to whitefly infestation. Dendrogram classification of time-points evidenced these differences which are also apparent on the PCA scores plot. ECU72 at pre-infestation consistently clusters away from the post-infestation time-points whilst in the susceptible variety COL2246, pre-infestation and early stages 0.5 and 1 dpi tend to group together (Figs. 2, 6a and 7a). This pattern of response in ECU72 may be associated with a primed mechanism that rapidly triggers a response to the presence of the whitefly [37–39].

The analysis of the time-series identified those metabolites changing with time that could be linked to the biotic stress response. Although 52 metabolites significantly changed with time, only 16 were shared by both genotypes, 6 were exclusively altered in COL2246 and a variation of 40 metabolites in ECU72 were associated with infestation time. The temporal variation observed in the susceptible variety presented a typical positive and negative linear tendency, i.e., metabolites gradually increasing and decreasing as infestation progressed. For example, the cyanogenic glycoside lotaustralin decreased with time as its anitrile by-products increased, which corresponds to the well-known toxicity mechanism described in cassava [40, 41]. However, in the resistant variety ECU72 the metabolites that significantly changed with time seem to follow a more complex pattern with subgroups of compounds presenting sudden rises or falls in abundance at certain time-points of the infestation that could be linked to biochemical events associated with the presence of different whitefly developmental stages. For example, flavonoids and malate and quinate esters of *p*-coumaric acids in clusters 2 and 5 of Fig. 7b displayed a rapid increase at 14 dpi that could have been triggered by the feeding activity of emerging nymphs.

#### Implications for the future breeding of whitefly resistance in cassava

There are numerous strategies to breed for elite germplasm to tackle emerging societal and environmental concerns. However, Marker Assisted Selection (MAS) remains the most rational or predominant approach presently used by the private plant breeding industry.

Although more closely related accessions can be used as donors during more refined trait breeding to maximise additive effects or heterosis, the necessity for natural variation is paramount. In the present study, we have exploited natural diversity present in Latin American germplasm for whitefly resistance/tolerance in cassava [8, 10, 42]. Concurrently, a susceptible and resistant accession has been revealed. These now potentially represent well characterised parental material from which segregating populations can be prepared, eventually leading to fixed marker defining regions that can be utilised to breed for the whitefly resistance trait. In the present example a number of differentiating metabolites have been identified to provide a specific chemical signature. These features have the potential to act as quantitative trait markers. The robustness of these features can now be tested under different environmental conditions or in the presence of different backgrounds which can confer epistatic effects. In addition, the metabolites associated with resistance could act as predictive markers during the construction of pre-breeding populations, where phenotypic parameters may not be pronounced, due to inadequate gene dosage.

## Conclusions

This study has used metabolomics to characterise a South American derived accession of cassava that is resistant to whitefly infestation in comparison to a geographically related susceptible accession. Our hierarchical approach to metabolomics was effective in rapidly capturing molecular features that differentiated the resistant chemotypes, in the presence and absence of infestation. Metabolite profiling was then used to confirm the changes in the metabolite pools characterising the resistant variety. Differences in metabolite profiles of both resistant and susceptible accessions ECU72 and COL2246 were detected consistently throughout the course of the whitefly infestation and also at pre-infestation time. Thus, it was concluded that the whitefly-resistance phenotype observed in the cassava accession ECU72 is associated to an antixenosis strategy based on reinforcement of cell wall. This hypothesis was based on the most significant chemical features differentiating both accessions being derivatives from biosynthetic intermediates of monolignols, preferentially accumulating in the susceptible variety compared to the higher deposition of lignin in the resistant variety. In addition, it was determined that the metabolomes of the susceptible and resistant accessions respond differently during whitefly’s infestation; the response to infestation is different between accessions.

These findings provide valuable insights into the underlying biochemical mechanism(s) associated with the resistance phenotypes; while providing characterised parental materials for future breeding programmes directed towards conferring whitefly resistance into staple crops in developing countries.

## Methods

### Plant material

In the tissue culture lab of the Cassava Genetics Program at CIAT, seedlings of each *Manihot esculenta* (Crantz) genotype COL2246 (whitefly-susceptible check) and ECU72 (whitefly-resistant check) from CIAT’s genebank collection were multiplied in vitro. Plants from in vitro culture, 8–10 weeks old, were planted in pots with sterile soil in a ratio of 3:1 sand to black soil (no clay topsoil) and kept in the glasshouse at 30 °C and 50–60% relative humidity [8]. CIAT’s genebank operates within the framework of the International Treaty on Plant Genetic Resources for Food and Agriculture (ITPGRFA) (http://www.fao.org/plant-treaty/overview/en/). Information related to original source of genotypes, characterisation and identification is stored in CIAT’s genebank and provided in the present manuscript as Additional file 15: Table S10.

### Infestation bioassays using *Aleurotrachelus socialis* (Bondar)

The *A. socialis* colony was raised on cassava genotype *Manihot esculenta* var. COL1468 as the host [8].

Non-choice experiments using a completely randomized design were used to challenge cassava genotypes ECU72 (whitefly (WF)-resistant) and COL2246 (WF-susceptible) [8, 10]. Each plant was put into individual cylindrical (1 m height × 30 cm diameter) mesh cages and 100 male and 100 female adults of *A. socialis* were released into each cage. Oviposition of adults was allowed for 72 h and plants were moved thereafter to a greenhouse free of whiteflies to allow progression of the whitefly life cycle. Five plants per genotype and time-point were used and three independent infestation trials were conducted, i.e. three biological replicates. Whole infested leaves carrying eggs or nymphs were collected from all plants at each time-point: zero hours post-infestation (T0), 12 hpi, (T1), 24 hpi (T2), 7 days post-infestation (T3), 14 dpi (T4), and 22 dpi (T5). Whiteflies from collected leaves were counted using a microscope and leaves were fast frozen in liquid nitrogen immediately after and stored at − 80 °C until lyophilization.

### Metabolites extraction

Freeze-dried material was ground to a fine powder by using tissue disruptor TissueRuptor (Qiagen) and aliquots of 10 mg used for metabolites extraction. Extractions were carried out in 50% methanol (1 h, shaking, room temperature) and 1 volume of chloroform was then added. Polar and non-polar metabolites were collected from methanolic epiphase and organic hypophase, respectively, after centrifugation. Both polar and non-polar extracts were filtered with 0.45 μm nylon membranes and 0.2 μm PTFE membranes prior to analysis. Quality control samples were prepared by pooling 10 mg from each sample and 3 technical replicates per sample were run on the different analytical platforms. A quality control sample and a blank of extraction (empty tube) were included every 25 analytical runs.

### LC-MS metabolite profiling

Polar extracts were analysed by LC-MS using a MAXIS UHR-Q-TOF mass spectrometer (Bruker Daltonics) and electrospray ion (ESI) source in negative mode. Ion source conditions were as follows: dry gas at 8 L/min, capillary 3500 V, end plate at − 500 V, vaporizing temperature was 195 °C and nebulizer was 1.3 Bar. Mass spectra were recorded in full scan mode from 100 to 1200 m/z range. A separate batch of analysis in MS/MS mode was also performed to facilitate chemical features fragmentation and structure characterization. Hence, a maximum of 4 ions above an intensity threshold of 1000 counts in every cycle were selected for fragmentation in a data-dependent manner by collision induced dissociation (CID). Separation of metabolites prior to MS detection was carried out using a UHPLC UltiMate 3000 equipped with a PDA detector (Dionex Softron). Chromatographic separations were performed in an YMC-UltraHTPro C18 2 μm column (100 × 2 mm i.d.) using 10% acetonitrile in water (A) and acetonitrile (B) as mobile phases in gradient mode, both containing 0.1% formic acid. These solvents were used in a gradient mode starting at 100% (A), held for 1 min, then stepped to 65% (A) in 17 min, followed by a linear gradient over 12 min to 0% (A). A 5 min washing and re-equilibration time was included in the gradient program. The flow rate used was 0.2 ml/min and the injection volume was 5 μl. Samples were spiked with genistein as an internal standard (0.01 mg/ml in the vial).

For the purpose of structural characterization, some of the extracts were also analysed by reversed phase LC-ESI-Fourier Transform-Ion Cyclotron Resonance (FT-ICR)-MS in negative ionization mode using an Accela UHPLC hyphenated to an LTQ FT Ultra (Thermo Electron Corporation, Bremen, Germany). Conditions were as mentioned in [43].

### GC-MS metabolite profiling

Components of intermediary/primary metabolism were mostly covered by gas chromatography coupled to an electron impact-single quadrupole mass spectrometer. Ten μl of the polar extract was spiked with 10 μl of the internal standard solution (1 mg/ml of deuterated succinic acid in methanol) and dried under vacuum (Genevac EZ.27). Dried extracts were derivatised to their methoxymated and silylated forms and analysed by GC-MS according to previous publication [44]. Similarly, 400 μl of the chloroform extract (non-polar phase) were spiked with 5 μl of internal standard (1 mg/ml of 5-α-cholestan-3-ol in chloroform) and dried under vacuum (Genevac EZ.27). Derivatisation of non-polar metabolites proceeded as above and temperature gradient on the GC-MS was adapted to optimize detection of terpenoids [45]. Raw data files generated from GC-MS analysis of non-polar and polar extracts are included as Additional files 13 and 14, respectively.

### Global profiling

Peak alignment, peak-picking and adducts grouping was performed on LC-MS netCDF raw data files using the R package metaMS [46]. The output was a matrix containing the intensity of each identified feature per sample and grouped in peak cluster (PC) groups, i.e., co-eluting groups of features that could arise from the same compound (isotopes, adducts, in-source fragments, etc.). GC-MS raw files were processed as in [44] using AMDIS version 2.71. Blank subtraction and batch correction using quality control (QC) samples were also performed on both LC-MS and GC-MS data and levels of metabolites were quantified relative to their corresponding internal standard.

### Targeted analysis

Annotation and identification of features detected under either the LC-MS and/or the GC-MS platforms enabled the creation of cassava-specific metabolite libraries based on the analysis of the MS fragmentation pattern and chemical formula generated from accurate mass. Electron impact (EI) MS spectra from GC-MS were interrogated in the NIST 17 library and Golm Metabolome Database (GMD) as described in [44]. Chemical formula generated from UHR-Q-TOF (LC-MS) accurate mass of parent ions, fragments and neutral losses obtained from CID MS spectra were searched in Chemspider and ChEBI databases enabling manual characterisation of compounds. In addition, structural validation was also obtained using an in-house FT-ICR-MS-based spectral database [43]. Bruker Compass DataAnalysis software v4.1 was used to calculate chemical formula from measured m/z values and neutral losses between parent ion and fragments. Chromatographic properties, analytical platform, and additional UV/Visible spectral information were also incorporated in the identification workflow.

### Data analysis strategy and statistical analysis

Quality and general overview of data was revised using principal component analysis (SIMCA v15, Umetrics). Data matrices were analysed using two strategies: (i) By pair-wise comparisons of genotypes per time-point using multiple t-test comparisons corrected by Holm-Sidak post-hoc test (α = 0.05); (ii) by investigating how each variety responds to the infestation of the whitefly by using analysis of variance (ANOVA) and multiple mean comparison Tukey HSD, Bonferroni or Dunnett post-hoc tests using pre-infestation time-point (T0) as control. Statistical analysis and graphs were performed using the XLSTAT, GraphPad Prism 7 and MetaboAnalyst online platform.

Results were painted over bespoken pathways constructed from KEGG and *Manihot esculenta*-PlantCyc dedicated databases and literature references.

### Analysis of lignin

Protein-free cell wall extracts were prepared following the protocol of [47] and 20 mg of this extract was used for lignin analysis. Lignin quantification was performed using the optimised acetyl bromide assay described in [47] and lignin monomeric composition and cross linked components were analysed by using the standard thioacidolysis and mild-alkali methods described in [48, 49].

## Supplementary information

**Additional file 1: Figure S1.** Egg counting of non-choice experiment and statistical analysis.

**Additional file 2: Table S1.** Fold-change of metabolites characterized by LC-MS and GC-MS in COL2246 and ECU72 and sorted by (**a**) analytical platform or (**b**) chemical class. Ratios defined as COL2246/ECU72 are highlighted in bold when significant (p < 0.05).

**Additional file 3: Table S2.** LC-MS untargeted data matrix sorted by retention time and obtained from global profiling data analysis as described in the Methods section.

**Additional file 4: Figure S2.** Principal component analysis of GC-MS analysis of non-polar extracts. (A) Score and (B) loadings plot of components 1 and 2. Collection times during infestation were defined by the following symbols: **◯** 0 days post-infestation (T0); ▼ 0.5 day (12 h) post-infestation (T1); ▲ 1 day post-infestation (T2); ■ 7 days post-infestation (T3); ✦ 14 days post-infestation (T4) and ★ 22 days post-infestation (T5). Principal component analysis plots were performed using Simca software and pareto-scaling method. Averaged biological and technical replicates are presented to facilitate visualisation.

**Additional file 5: Figure S3.** Principal component analysis of GC-MS analysis of polar extracts. (A) Score and (B) loadings plot of components 1 and 2. Collection times during infestation were defined by the following symbols: **◯** 0 days post-infestation (T0); ▼ 0.5 day (12 h) post-infestation (T1); ▲ 1 day post-infestation (T2); ■ 7 days post-infestation (T3); ✦ 14 days post-infestation (T4) and ★ 22 days post-infestation (T5). Principal component analysis plots were performed using Simca software and pareto-scaling method. Averaged biological and technical replicates are presented to facilitate visualisation.

**Additional file 6: Table S3.** Characterization and annotation of compounds detected under LC-MS analysis based on the in-source and CID fragmentation and chemical formula generated from the accurate mass measured.

**Additional file 7: Table S4.** Results of ANOVA analysis of COL2246 time-series.

**Additional file 8: Table S5.** Results of ANOVA analysis of ECU72 time-series.

**Additional file 9: Figure S4.** Comparative variation pattern of leaf metabolites during whitefly infestation and leaf development (untreated). Only core metabolites changing in both COL2246 and ECU72 identified from Fig. 4b are compared.

**Additional file 10: Table S6.** Results of analysis of lignin of cassava leaves. Quantification and detection of hydroxycinnamic acids (HCAs) and lignin monomers after alkali and acid hydrolysis was performed by GC-MS as described in the Methods section.

**Additional file 11: Table S7.** Quantification of phenylpropanoid subfamilies hydroxycinnamic acids, flavonoids, lignans and lignin oligomers in COL2246 and ECU72

**Additional file 12: Figure S5.** Quantification of total phenylpropanoids or phenylalanine derived compounds and subfamilies in COL2246 and ECU72 at each time-point of infestation. Left-hand columns indicate absolute amounts (μg/g DW) of each chemical class and right-hand columns illustrate the relative amount (%) of each chemical family respective the total amount of phenylpropanoids. Red arrow indicates the level of fold-change (fc) increase . See Additional file 11: Table S7

**Additional file 13: Table S8.** GC-MS raw data matrix sorted by retention time obtained from analysis of non-polar.

**Additional fille 14: Table S9.** GC-MS raw data matrix sorted by retention time obtained from analysis of non-polar.

**Additional file 15: Table S10.** Passport data of accessions COL2246 and ECU72 from CIAT’s genebank

## Data Availability

The materials used during the current study will be available upon request to corresponding author p.fraser@rhul.ac.uk. Raw data sets generated from the LC-MS and GC-MS analysis of the plant material used in the present study are available in this published article as additional information files 3, 13 and 14 (.xlsx). All data analysed during this study are included in this published article as additional information files.

## Abbreviations

ACMV: African cassava mosaic virus
CBSV: Cassava brown streak virus
CID: Collision induced dissociation
Dpi: Days post-infestation
DW: Dry weight
EI: Electron impact
GC-MS: Gas chromatography-mass spectrometry
Hpi: Hours post-infestation
LC-MS: Liquid chromatography-mass spectrometry
PCA: Principal component analysis
UHPLC: Ultra-high performance liquid chromatography
WF: Whitefly
