# Supplementary figures and images for: A metabolomics characterisation of natural variation in the resistance of cassava to whitefly
Source: BMC Plant Biol. 2019 Nov 27;19:518. doi: 10.1186/s12870-019-2107-1 (PMC6882011; doi:10.1186/s12870-019-2107-1)

## Slide 1
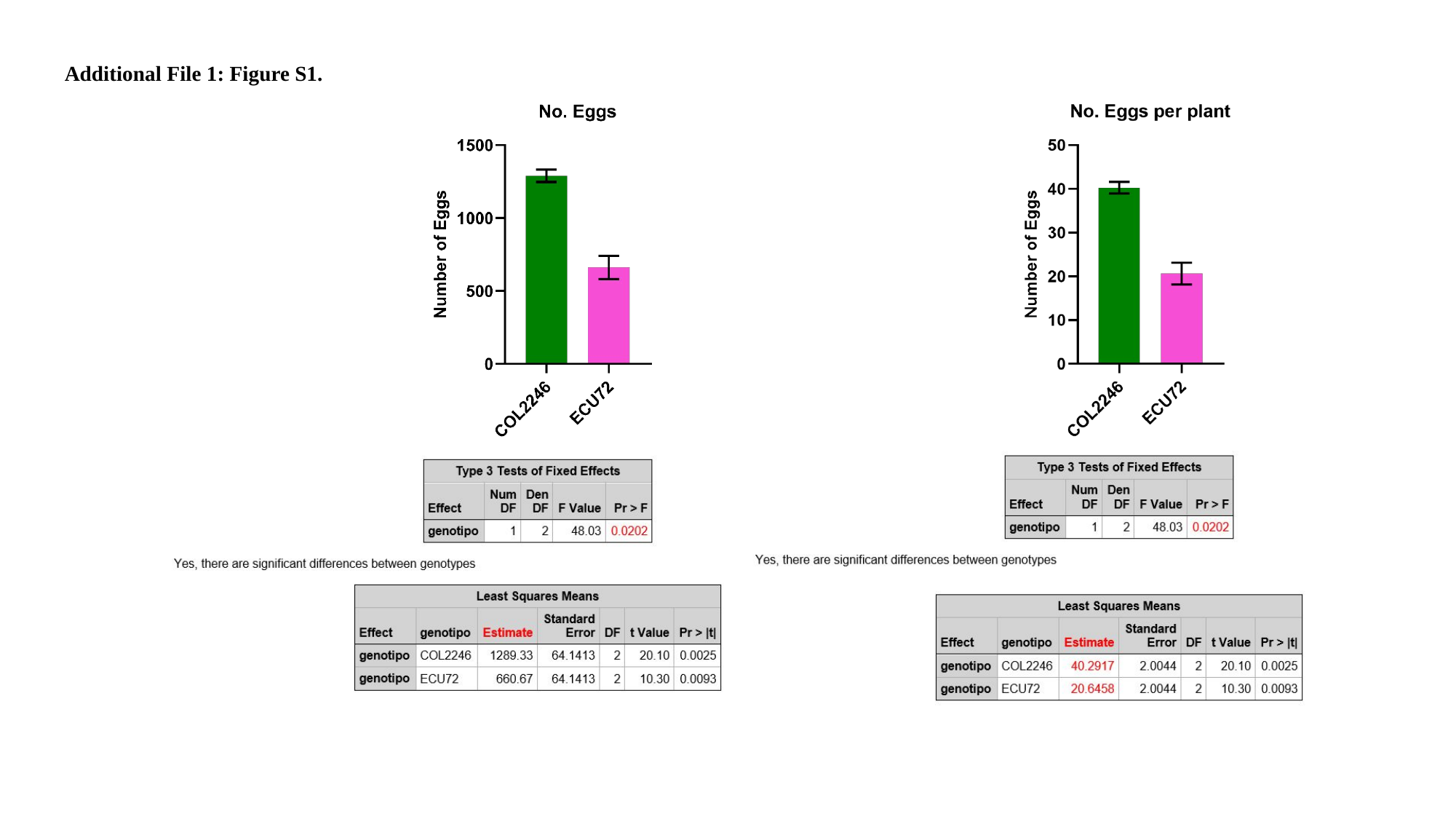

Additional File 1: Figure S1.

Supplement: Supplementary file 1 — Additional file 1: Figure S1. Egg counting of non-choice experiment and statistical analysis. [file 12870_2019_2107_MOESM1_ESM.pptx]

## Slide 1
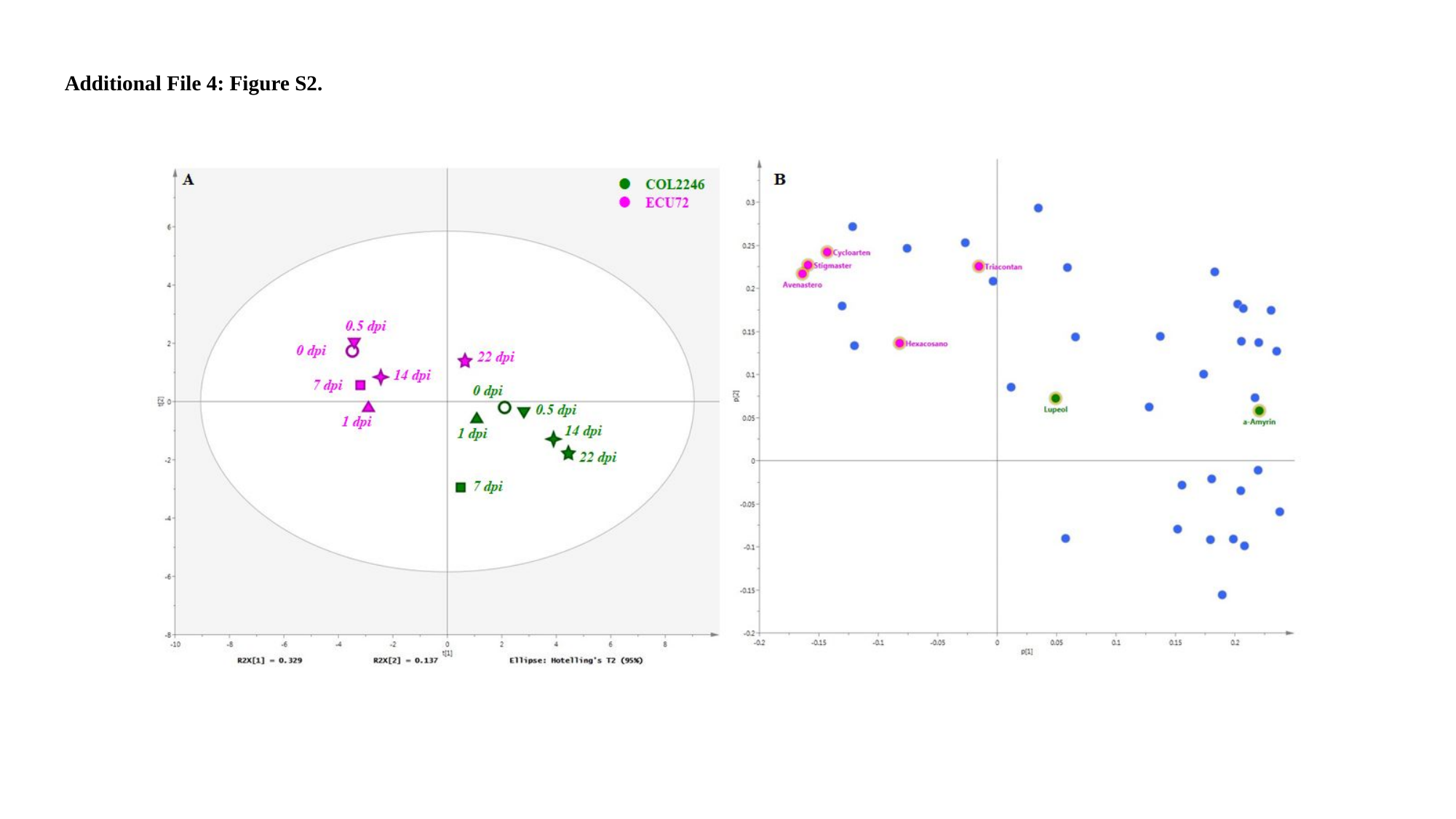

Additional File 4: Figure S2.

Supplement: Supplementary file 4 — Additional file 4: Figure S2. Principal component analysis of GC-MS analysis of non-polar extracts. (A) Score and (B) loadings plot of components 1 and 2. Collection times during infestation were defined by the following symbols: ◯ 0 days post-infestation (T0); ▼ 0.5 day (12 h) post-infestation (T1); ▲ 1 day post-infestation (T2); ■ 7 days post-infestation (T3); ✦ 14 days post-infestation (T4) and ★ 22 days post-infestation (T5). Principal component analysis plots were performed using Simca software and pareto-scaling method. Averaged biological and technical replicates are presented to facilitate visualisation. [file 12870_2019_2107_MOESM4_ESM.pptx]

## Slide 1
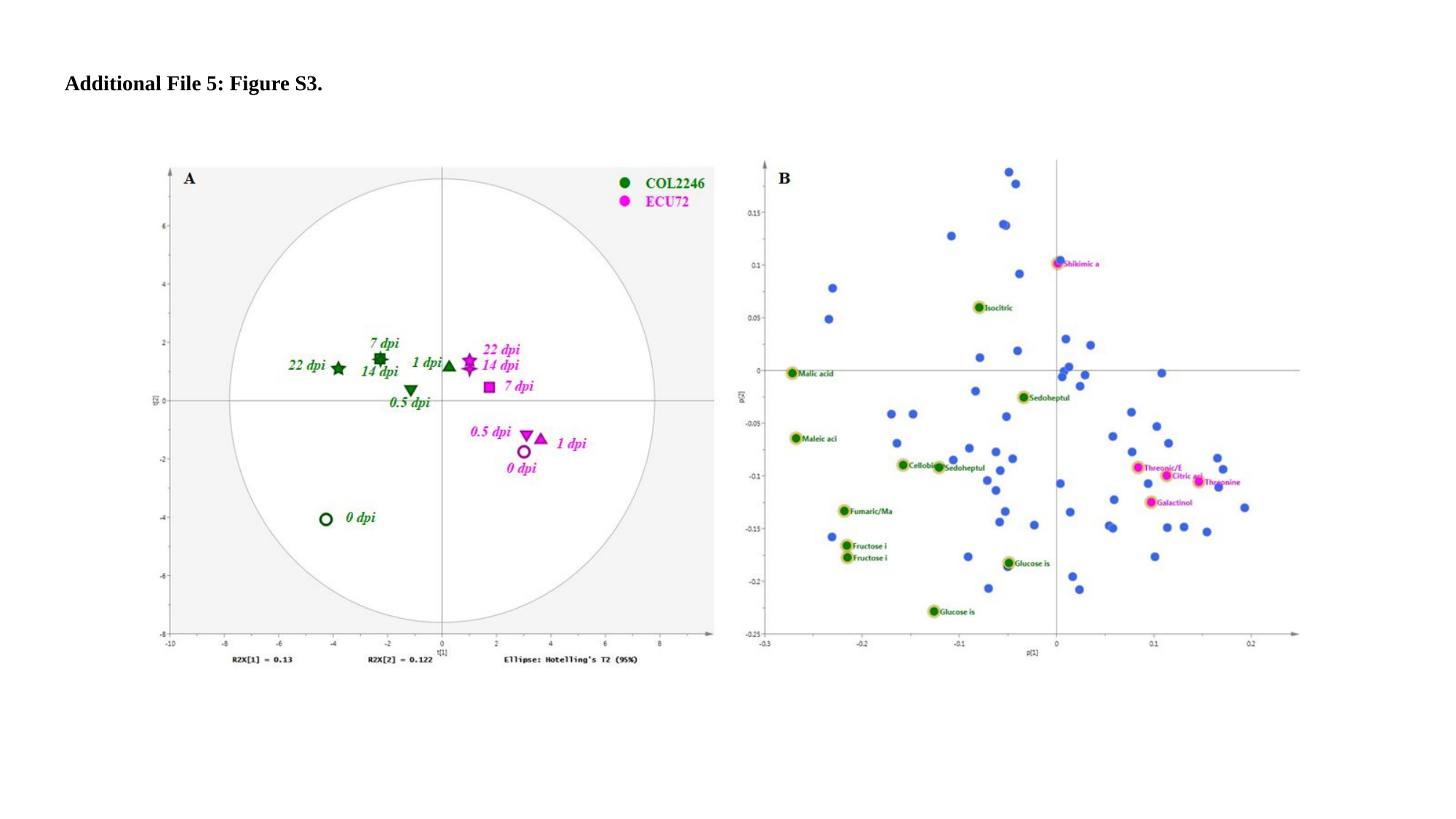

Additional File 5: Figure S3.

Supplement: Supplementary file 5 — Additional file 5: Figure S3. Principal component analysis of GC-MS analysis of polar extracts. (A) Score and (B) loadings plot of components 1 and 2. Collection times during infestation were defined by the following symbols: ◯ 0 days post-infestation (T0); ▼ 0.5 day (12 h) post-infestation (T1); ▲ 1 day post-infestation (T2); ■ 7 days post-infestation (T3); ✦ 14 days post-infestation (T4) and ★ 22 days post-infestation (T5). Principal component analysis plots were performed using Simca software and pareto-scaling method. Averaged biological and technical replicates are presented to facilitate visualisation. [file 12870_2019_2107_MOESM5_ESM.pptx]

## Slide 1
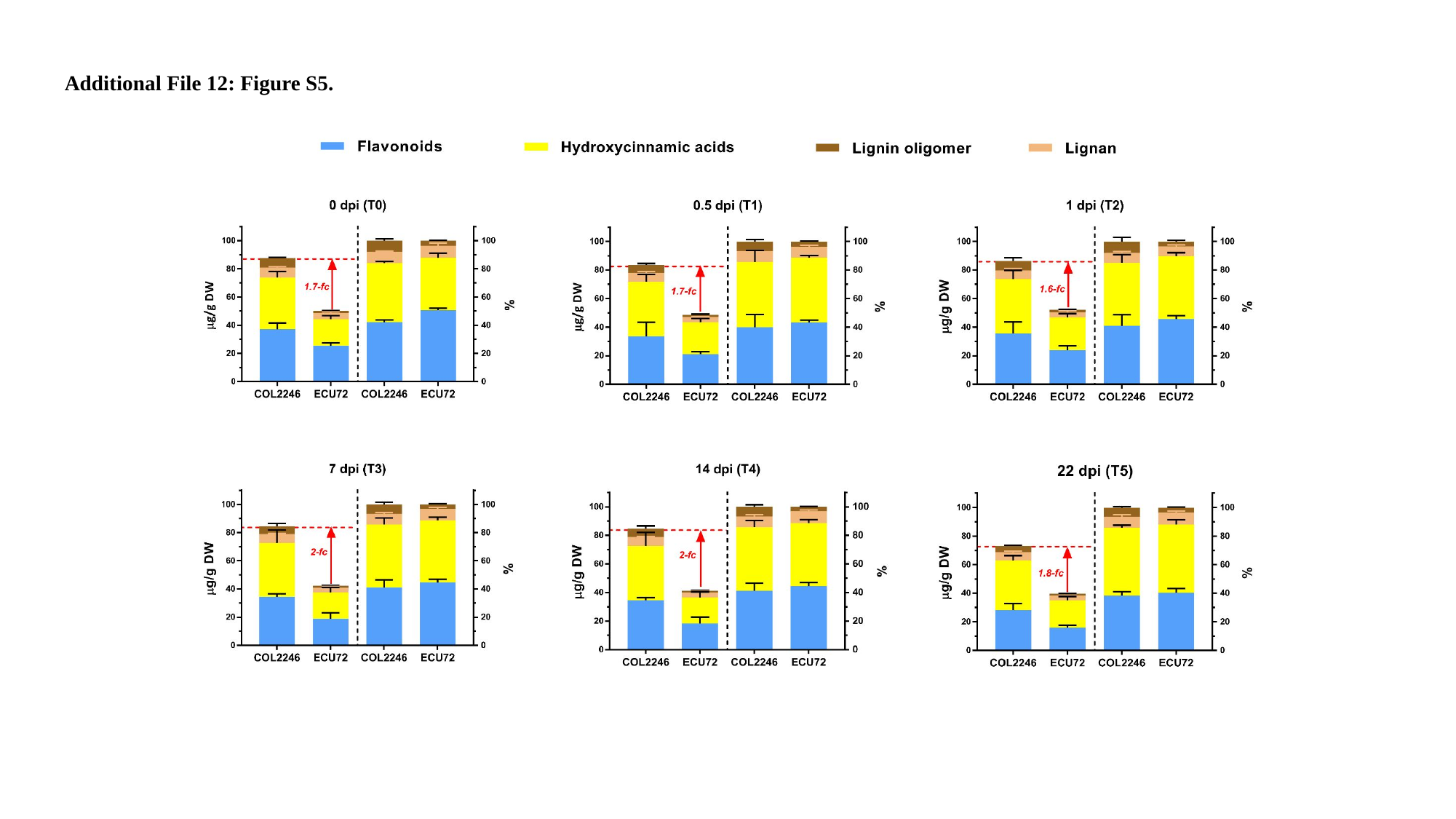

Additional File 12: Figure S5.

Supplement: Supplementary file 12 — Additional file 12: Figure S5. Quantification of total phenylpropanoids or phenylalanine derived compounds and subfamilies in COL2246 and ECU72 at each time-point of infestation. Left-hand columns indicate absolute amounts (μg/g DW) of each chemical class and right-hand columns illustrate the relative amount (%) of each chemical family respective the total amount of phenylpropanoids. Red arrow indicates the level of fold-change (fc) increase . See Additional file 11: Table S7 [file 12870_2019_2107_MOESM12_ESM.pptx]
