# Supplementary material for: A metabolomics characterisation of natural variation in the resistance of cassava to whitefly
Source: BMC Plant Biol. 2019 Nov 27;19:518. doi: 10.1186/s12870-019-2107-1 (PMC6882011; doi:10.1186/s12870-019-2107-1)
Supplement: Supplementary file 9 — Additional file 9: Figure S4. Comparative variation pattern of leaf metabolites during whitefly infestation and leaf development (untreated). Only core metabolites changing in both COL2246 and ECU72 identified from Fig. 4b are compared. [file 12870_2019_2107_MOESM9_ESM.pptx]

## Slide 1
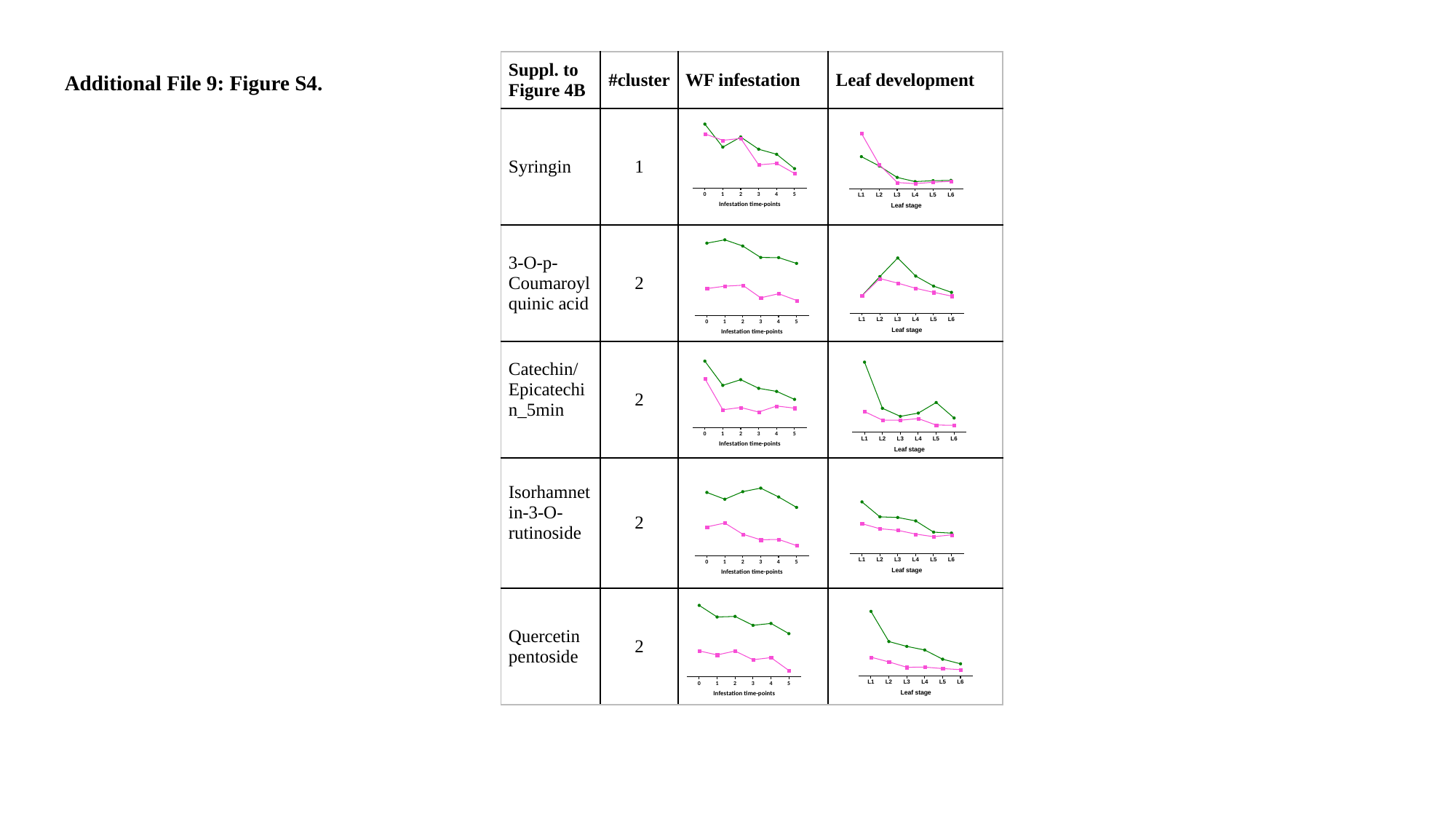

| Suppl. to Figure 4B | #cluster | WF infestation | Leaf development |
| --- | --- | --- | --- |
| Syringin | 1 | | |
| 3-O-p-Coumaroylquinic acid | 2 | | |
| Catechin/Epicatechin\_5min | 2 | | |
| Isorhamnetin-3-O-rutinoside | 2 | | |
| Quercetin pentoside | 2 | | |
Additional File 9: Figure S4.

## Slide 2
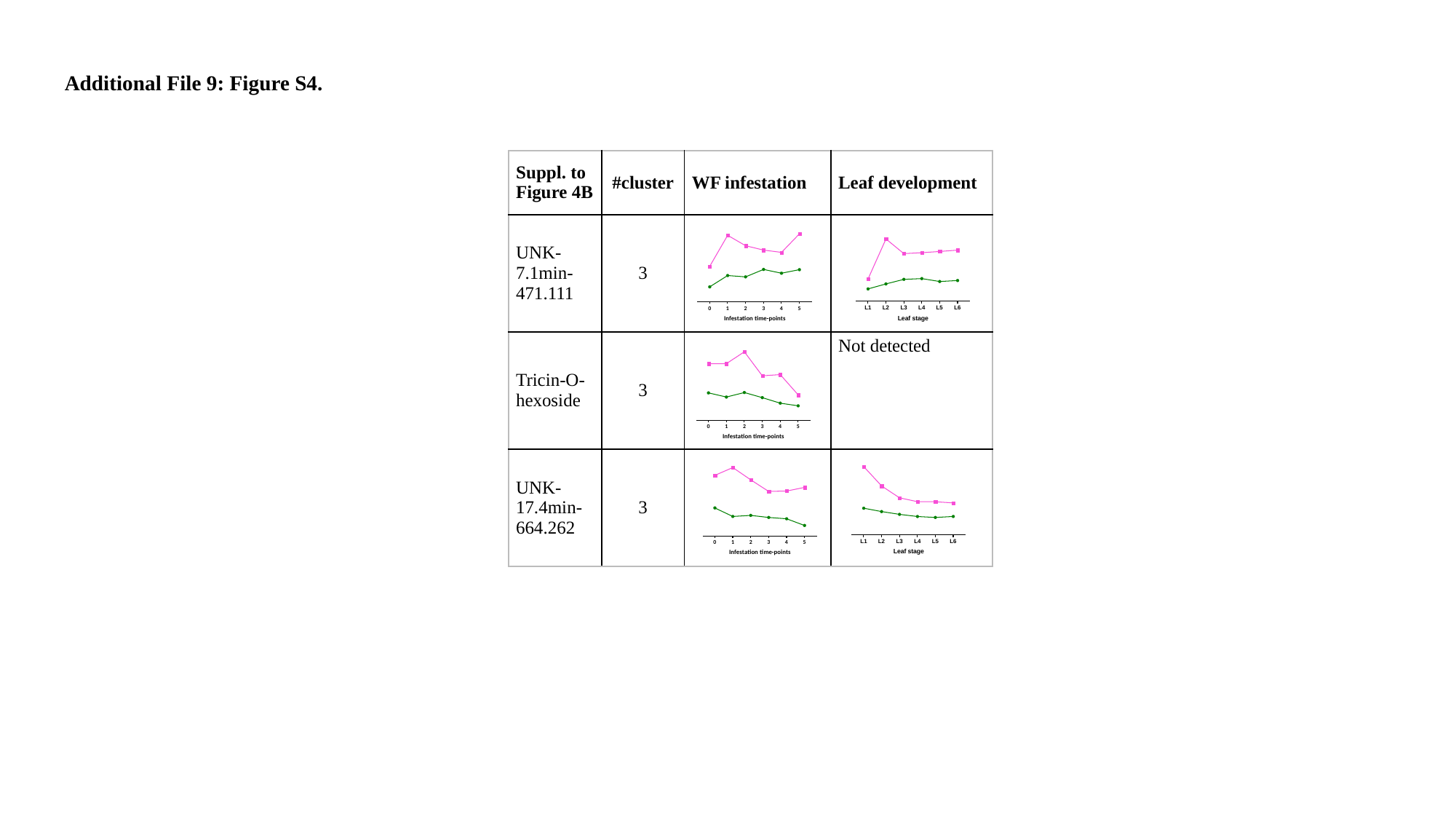

Additional File 9: Figure S4.
| Suppl. to Figure 4B | #cluster | WF infestation | Leaf development |
| --- | --- | --- | --- |
| UNK-7.1min-471.111 | 3 | | |
| Tricin-O-hexoside | 3 | | Not detected |
| UNK-17.4min-664.262 | 3 | | |

## Slide 3
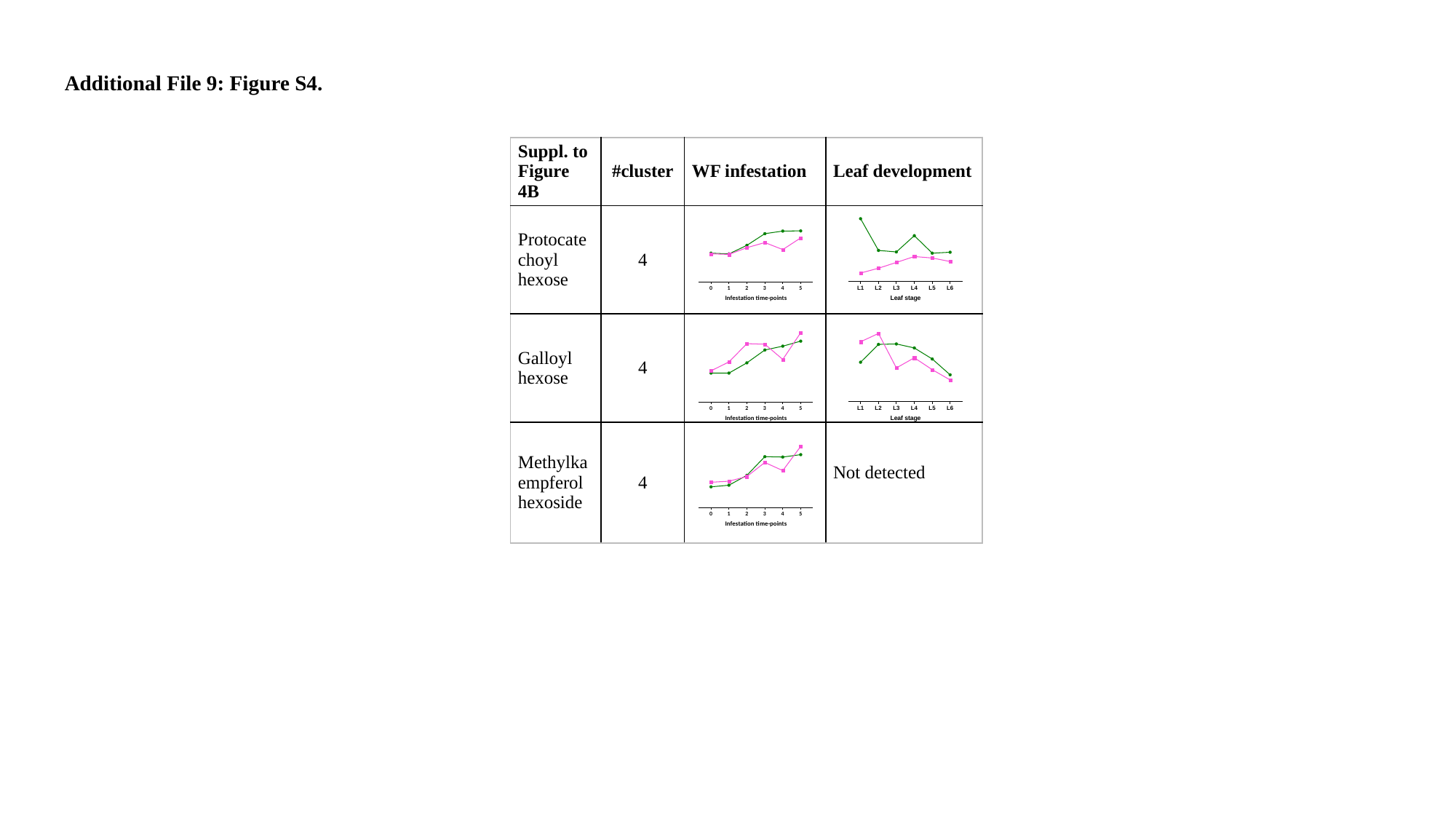

Additional File 9: Figure S4.
| Suppl. to Figure 4B | #cluster | WF infestation | Leaf development |
| --- | --- | --- | --- |
| Protocatechoyl hexose | 4 | | |
| Galloyl hexose | 4 | | |
| Methylkaempferol hexoside | 4 | | Not detected |

## Slide 4
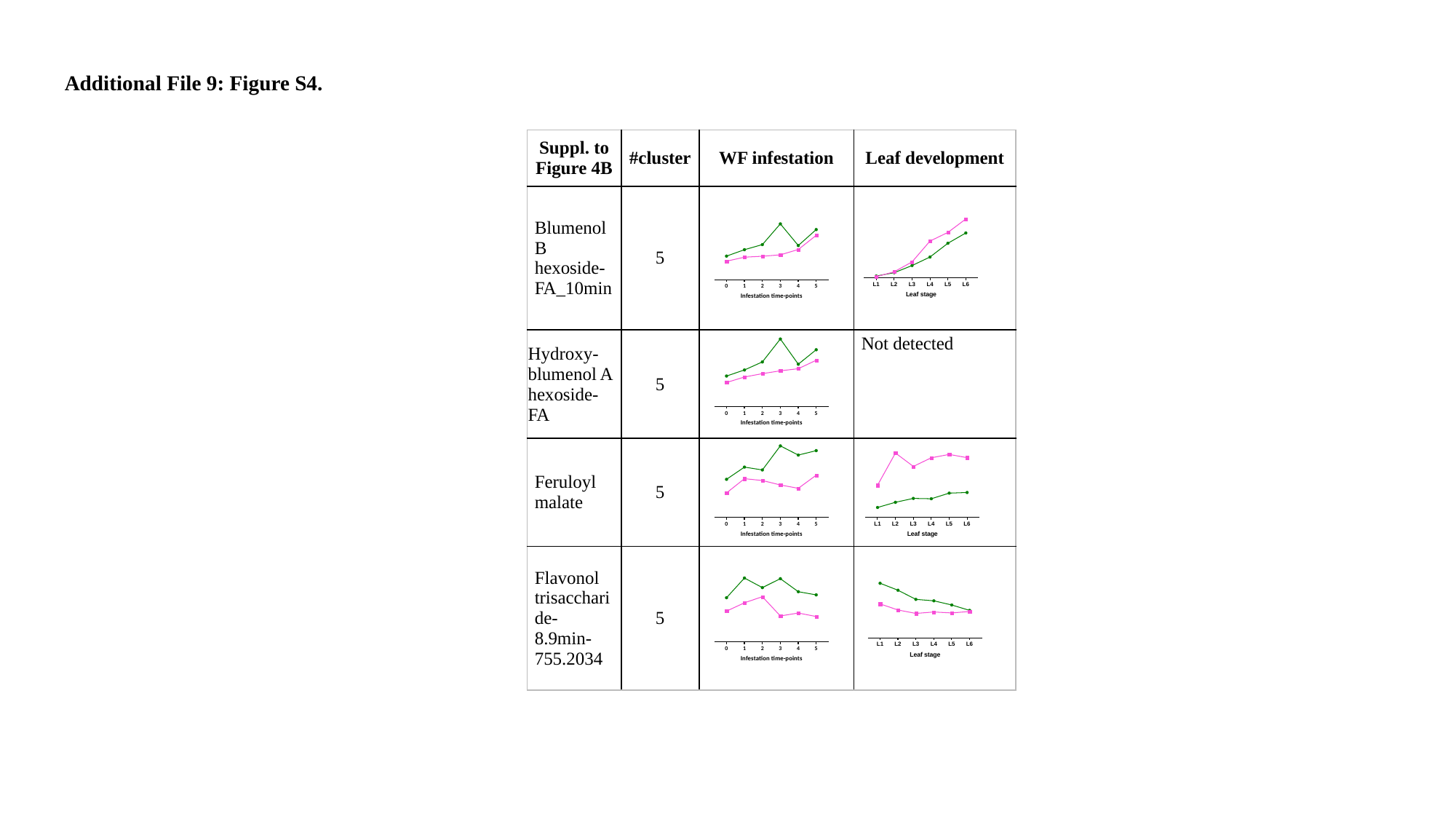

Additional File 9: Figure S4.
| Suppl. to Figure 4B | #cluster | WF infestation | Leaf development |
| --- | --- | --- | --- |
| Blumenol B hexoside-FA\_10min | 5 | | |
| Hydroxy-blumenol A hexoside-FA | 5 | | Not detected |
| Feruloyl malate | 5 | | |
| Flavonol trisaccharide-8.9min-755.2034 | 5 | | |
